# Supplementary material for: Awareness of fifth metatarsal stress fractures among soccer coaches in Japan: A cross-sectional study
Source: PLoS One. 2024 May 14;19(5):e0277582. doi: 10.1371/journal.pone.0277582 (PMC11093340; doi:10.1371/journal.pone.0277582)
Supplement: Attachment — Submitted filename: Response_to_Reviewers_TM-5_revised.docx [file pone.0277582.s002.docx]

| **Reviewer comments** | **Answers** |
| --- | --- |
| Reviewer #1: I would recommend few corrections or some of my doubts need comments before the final publication: | Thank you for your comments. We have answered them below. |
| 1. The authors wrote that they examined the knowledge, however they use the word "awarness" all the time. I do not agree that those words can be used interchangably. So, what was evaluated? | Thank you for this question. Considering the study’s purpose and the questionnaire utilised, we believe “awareness” is a more suitable term. Therefore, we changed the term “*knowledge*” to “*awareness*” throughout the revised manuscript. |
| 2. How was the questionnaire prepared, the answers....to check knowledge? It is ood to use Likert scale or in some other form of raiting....e.g. to evaluate it. Other way, it can be only said how many people answer on sth. but how deep they knew the meaning of the question? It also influance their awarness.... | Thank you for this question. We have added some comments in Materials and Methods to explain how the questionnaire was developed, as follows: “*A computer-based survey was created following a literature review of the injury, treatment, and prevention of MT-5 and a discussion with experienced physical therapists, sports doctors, and sports scientists who were familiar with MT-5. This study’s main interest was to understand differences in the awareness about the injury, treatment, and prevention of MT-5 by license level and coaching environment. Hence, the questionnaire was designed with an emphasis on how many coaches were aware of this information rather than the depth of their knowledge of MT-5.”(p4 lines 73–79).*  In addition, we realise that this was a limitation of the study. Hence, we have added some comments on the study’s limitations as follows: “*Second, the study’s questionnaire was designed to investigate the awareness of MT-5, not the depth of respondents’ understanding of MT-5. Therefore, there may be differences in the level of understanding even among respondents aware of it.” (p10, lines 192–194)* |
| 3. In the Abstract the sample size os 117, but in Methods is still 150. Please, correct. | Thank you for pointing this out. We have revised the abstract as follows: “*A total of 150 soccer coaches were invited for an original online survey*” (*p2 lines 21–22*) and *“Data from 117 coaches were analysed.” (p2 line 25)* |
| 4. Some references are quite old...Ref.1 | We have retained reference 1 in the revised manuscript because it defined the “Torg classification.” However, we have changed the place of the reference number in the manuscript as follows: “*In particular, zones II and III in the MT-5 Torg classification [1]…” (p3 line 43)*  Reference 5 (Kavanaugh 1978) has been deleted from the manuscript since reference 5 (Kane 2015) can suffice. (*p3 line 52*)*.* |
|  | In addition, we thought it would be better to add the test-retest reliability results. Therefore, we have included an additional comment on the statistical analysis as follows: “*The intraclass correlation coefficients (ICC) and kappa statistics were used to measure the degree of agreement between responses from 20 participants to confirm the test-retest reliability of the questionnaire*” (*p5 lines 95–98*) We have also revised the results section as follows: “*The ICC and kappa values for the test-retest reliability were excellent (ICC range = 0.88 - 0.98) and moderate to almost perfect (k range = 0.57–0.88), respectively.” (p6 lines 115–117)* |
