# Supplementary material for: Awareness of fifth metatarsal stress fractures among soccer coaches in Japan: A cross-sectional study
Source: PLoS One. 2024 May 14;19(5):e0277582. doi: 10.1371/journal.pone.0277582 (PMC11093340; doi:10.1371/journal.pone.0277582)
Supplement: Attachment — Submitted filename: Response_to_Reviewers_PROSone 0829.docx [file pone.0277582.s003.docx]

| Comments | Responses |
| --- | --- |
| Reviewer  Abstract: Line36-38: Suggest to rephrase the sentence- it was unclear | We have modified the text as follows: *“Our findings suggest the need for increased awareness of fifth metatarsal stress fractures and improved education of soccer coaches regarding injury prevention strategies.”* (lines 36–38) |
| Introduction Line 43-45: Suggest to rephrase the sentence to show that the common fracture is at the Zone II and III of the 5th MTB which also known as Jones fracture. | We have modified the text as follows: “*It frequently occurs at the zones II and III in the MT-5 Torg classification [1], which are located in the proximal shaft of the fifth metatarsal and known as Jones fractures [2]*.” (lines 44–45) |
| Line 46: To confirm reinjury is one of the factors for surgery? | For clarity, we have modified the text as follows: “*MT-5 mainly treated through surgery, as it is difficult to achieve bone union with conservative treatment due to the poor blood supply at the fifth metatarsal [3].*” (lines 45–47) |
| Line 54: immobilization of the lateral plantar- pathological or by brace? | We intended to mention the risk factors for MT-5. Thus, we have rephrased this sentence (Please check our response to the following question). |
| Line 55-56: suggest to rephrase sentence | We appreciate the reviewer’s question. We have modified the text as follows: *“In addition, a deficiency in 25-hydroxyvitamin D, which is an indicator of calcium metabolism [6], foot distraction tendencies during loading [7], limited range of motion of hip internal rotation [6], and toe grip muscle weakness [7] have been reported to be associated with the occurrence of MT-5.*” (lines 54–57) |
| Line 64-66: is the objective of the study? | Yes, it is. We have modified the sentence to make the objective clearer, as follows: “*we conducted a questionnaire survey of soccer coaches affiliated with the Japan Football Association (JFA), and the purpose of this study was to investigate their awareness of MT-5 and its prevention and treatment*.” (lines 61–63) |
| Materials & Methods The methodology was not very clear. A new questionnaire was created. Validation of the questionnaire was based on the expert committee? | We appreciate the reviewer’s question. Yes, we made a new questionnaire based on literature review and expert opinions. However, we did not conduct a validation study yet for our new questionnaire. Therefore, we have modified the methodology, as follows: “*A computer-based survey was created following a literature review of the injury, treatment, and prevention of MT-5 and a discussion with experienced physical therapists, sports doctors, and sports scientists who were familiar with MT-5 to cover all the relevant content.*“ (lines 71–74).  In addition, we have added a limitation, as follows: “*Third, the content validity of the questionnaire was not confirmed through qualitative research, as suggested by a previous study [19]; however, we developed it based on literature review and expert opinions.*” (lines 202–204) |
| Test-retest reliability was done among the coaches? | Yes, test-retest reliability was done among the coaches. We have clarified this point in the Methods as follows: “*In addition, 20 participants among all responders across all coaching categories were randomly assigned to complete the survey a second time 1 week after completing their first survey to identify the reliability of the self-reported questionnaire.*” (lines 90–93) |
| Coaches for test-retest was also involved in the main study? | Yes, coaches for the test-retest analysis were also involved in the main study. We have clarified this point in the methods, as follows: “*These analyses were performed for all responders, while the data from the first survey was employed for those who responded twice for the test-retest reliability.” (lines 102–104)* |
| Was the study applied for ethics clearance? | Yes, we obtained ethical clearance for this study, as mentioned in lines 109–111: “*This study was approved by the International University of Health and Welfare Ethics Review Board (approval number: 16-Io-203). All survey respondents provided written informed consent.*” |
| Result The result did not answer the objective of the study which want to study the coaches awareness of MT-5, its prevention and treatment. In the result- the correlation was described in detail; however, the prevention and treatment strategies were covered superficially. | We have modified the results, as follows: “*Regarding the treatment methods, 20.0% (3/15) of the unlicensed coaches, 0% (0/3) of those in D-level, 25.7% (9/25) in C-level, 20.7% (6/29) in B-level, 34.2% (13/38) in A-level, and 57.1% (4/7) in S-level. In the awareness of preventive training strategies, respondents who were aware of it were 26.7% (4/15) in unlicensed coach, 0% (0/3) in D-level, 28.0% (7/25) in C-level, 27.6% (8/29) in B-level, 34.2% (13/38) in A-level, and 57.1% (4/7) in S-level were aware of the treatment.* “ (lines 131–134)  In addition, we have added the following text to the Discussion: “*However, only 30% of the coaches implemented treatment and preventive training, although the higher the class of license, the greater the awareness of treatment and prevention strategies.”* (lines 155–157) |
| Discussion Good discussion, but suggest to include other related studies that involved 5th MTB stress fracture | We have added two references (reference 10 and 18) and supplemented the discussion, as follows: “*However, currently, coaches do not adequately address factors related to MT-5 using preventive interventions and rehabilitation, despite the significant time-loss from playing soccer associated with this injury [10].*” (lines 169–171), and “*To reduce the incidence of MT-5, doctors and physical therapists need to provide specialized and comprehensive programs regarding medical science and preventive training methods for coaches, given the multifactorial nature of the causes of MT-5 [6,7 15-18].*” (lines 190–192). |
| Suggest to provide the sample of the online survey as supplement | We appreciate the reviewer’s suggestion. As it was prepared in Japanese and we are currently considering adapting it to the English version, so it is difficult to provide it. Instead, we will respond sincerely to any inquiries about it. |
| 5MT- not sure if it is acceptable as acronym | MT-5 seems acceptable as an acronym of the fifth metatarsal fracture, as it has been used in many scientific journals. |
| Too many semicolon (;) May need to check the writing style | We have modified the text for proper use of semicolons, and the writing style has been checked by a professional English editing service. |
