# Supplementary material for: Awareness of fifth metatarsal stress fractures among soccer coaches in Japan: A cross-sectional study
Source: PLoS One. 2024 May 14;19(5):e0277582. doi: 10.1371/journal.pone.0277582 (PMC11093340; doi:10.1371/journal.pone.0277582)
Supplement: Attachment — Submitted filename: questions_and_answers_20240112.docx [file pone.0277582.s004.docx]

| Major revision  Material and method  • The authors have missed something crucial. The study fails to address content validity. Content validity is a crucial aspect of testing or measuring anything. | Thank you for your valuable comment. As you suggested, we did not confirm the content validity through qualitative research, as mentioned in the limitations section. However, we developed the questionnaire with input from relevant specialists who are familiar with MT-5.  While we acknowledge that this is one of the limitations in our study, we have included in the Materials and Methods section the following statement: “The final version of the questionnaire was established through consensus among all members to encompass all relevant aspects of MT-5.” (Lines 74-76) |
| --- | --- |
| Minor revision  Introduction Good introduction!  • lines 51-53: Please move the sentence to line 4 to emphasize the incidence. | Your valuable comment is greatly appreciated. Although it is not clear where your references to lines 51-53 and 4 are, we have relocated the statement “In injury surveys of European professional soccer players, the incidence of stress fractures was approximately 5% of all injuries, 78% of which were MT-5.” to immediately follow the first sentence. This adjustment aims to emphasize the incidence. (Lines 43-44) |
| • The prevalence of MT-5 is different in non-athletic men and women and athletes. Boutefnouchet et al (<https://doi.org/10.1177/1460408614525738> ). Suggest to add information about gender differences in the introduction. | Thank you for bringing this to our attention. In the Introduction, we have added the following statement: “with male athletes, in particular, being at greater risk than female athletes [6].” (Lines 53-54) |
| Material and method  • Line 80: What do the authors mean by sports doctors? If the authors mean sports medicine physician, suggest to rephrase it. | Thank you for addressing this issue. We have rephrased the sentence. (Line 74) |
| • Lines 93-97: Since one of the objectives of this article was to investigate awareness about MT-5 therapy, the authors neglected to mention it. | Thank you for raising this point. We have added “awareness of treatment methods and preventive training strategies for MT-5.” (Line 92) |
| • Please clarify how the authors assessed MT-5 treatment awareness in the method section. | I appreciate your attention to this matter. In relation to the following question, we have relocated the sentence from the Results section and included it in the Materials and Methods section with the following wording: “MT-5 prevention methods were assessed using a free-format question, while other items were assessed using multiple-choice questions.” (Lines 92-94) |
| Results  • One of the characteristics of the participants was their gender. The authors did not mention any results about it. Please add information about gender distribution. | Thank you for your important suggestion. We have added the statement: “We contacted 150 potential participants, and the data from 117 male respondents (average age: 39 ± 9 years, effective response rate: 78%) were analysed.” in the Results section. (Lines 117-118) |
| • Lines 130-131: “MT-5 prevention methods were assessed using a free-format question”. Suggest to move this sentence to the material and method section. | Thank you for your insightful suggestion. We have relocated this sentence to the Materials and Methods section and revised it as follows: “Regarding MT-5 prevention methods, the most frequent response was adjusting the training volume; other responses included trunk training, ankle tube training, and changing spike cleats.” (Lines 126-128) |
| • Table 1: Last row “preventive training”: the objective was to investigate the awareness of prevention, suggest to rewrite it. • Table 2 and table 3: The authors used “MT-5 recognition” in the title. Suggest to rephrase it to “MT-5 Awareness”. | Your meaningful comment is duly noted. We have made the recommended changes to Tables 1, 2, and 3. |
| • Please provide the sample of the online survey as supplement. | We value your perceptive comment.  As it was originally prepared in Japanese and we are currently in the process of adapting it into English, it is not readily available. Nevertheless, we will gladly respond sincerely to any inquiries regarding it. |
